# First-line sequential high-dose VIP chemotherapy with autologous transplantation for patients with primary mediastinal nonseminomatous germ cell tumours: a prospective trial

**DOI:** 10.1038/sj.bjc.6600999

**Published:** 2003-07-01

**Authors:** C Bokemeyer, N Schleucher, B Metzner, M Thomas, O Rick, H-J Schmoll, C Kollmannsberger, I Boehlke, L Kanz, J T Hartmann

**Affiliations:** 1 Tuebingen University Medical Center II, Department of Hematology, Oncology, Rheumatology and Immunology, Eberhard-Karls-University, Otfried-Mueller-Str. 10, 70276 Tuebingen, Germany; 2 Internal Medicine, West German Cancer Center, Essen, Germany; 3 Klinikum Oldenburg, Germany; 4 Department of Medicine, Hematology, Oncology, Respiratory Medicine, University of Münster, Germany; 5 Charite Campus Mitte, Department of Hematology and Oncology, Berlin, Germany; 6 Department of Hematology and Oncology, University of Halle, Germany

**Keywords:** extragonadal germ cell tumours, mediastinal primary, high-dose chemotherapy, autologous transplantation, nonseminomatous histology

## Abstract

To determine the efficacy of first-line sequential high-dose VIP chemotherapy (HD-VIP) in patients with primary mediastinal nonseminomatous germ cell tumours (GCT), 28 patients were enrolled on a German multicentre trial. High-Dose VIP chemotherapy consisted of 3–4 cycles of dose-intensive etoposide and ifosfamide plus cisplatin, q22days, each cycle followed by autologous peripheral blood stem cell transplantation plus granulocyte-colony stimulating factor (G-CSF) support. One cycle of standard-dose VIP was applied to harvest peripheral blood stem cells. Ten patients had mediastinal involvement as the only manifestation (36 %), 18 of 28 patients had additional metastatic sites, such as lung (*n*=17; 61%), liver (*n*=7; 25%), bone (*n*=5; 18%), lymph nodes (*n*=3; 11%) and CNS (*n*=3; 11%). Median follow-up was 43 months (range, 7–113) for all patients and 52 months (range, 22–113) for surviving patients. Nineteen of 28 patients obtained a disease-free status; 11 with HD-VIP alone and eight with adjunctive surgery. In addition, one of the four patients with marker negative partial remission after HD-VIP without resection of residual masses is currently alive. Two patients developed recurrence of GCT or teratoma. Two patients have died due to an associated haematologic disorder. The 2-year progression-free survival and overall survival rates are 64 and 68%, respectively. This report represents a subgroup analysis of 28 patients with mediastinal nonsemina within the German first-line study for ‘poor prognosis’ GCT. Compared to data of an international database analysis including 253 patients with mediastinal nonseminoma treated with conventional chemotherapy, the results may indicate that HD-VIP results in an approximately 15% survival improvement.

Testicular germ cell cancer is the most common malignancy in men aged between 15 and 35 years (Hartmann *et al*, 1999, Bosl and Motzer, 1997). An important subset of germ cell tumours (GCT), approximately 5–7%, is of extragonadal origin (Collins and Pugh, 1964). In adults, these tumours most commonly arise in the midline of the body, particularly in the retroperitoneum and the mediastinum. Conventional cisplatin-based chemotherapy has demonstrated activity in patients with extragonadal GCT, and long-term survival rates approach those of patients with advanced-stage metastatic gonadal GCT (Nichols *et al*, 1990b, Hidalgo *et al*, 1997). Primary nonseminomatous tumours of the mediastinum have a poor outcome with conventional chemotherapy and the presence of a mediastinal location defines the patient as ‘poor prognosis’ according to the *IGCCCG* classification irrespective of additional metastatic sites or elevated tumour marker concentrations (Ulbright *et al*, 1984; Toner *et al*, 1991; Bukowski *et al*, 1993; Childs *et al*, 1993; Harding *et al*, 1993; Saxman *et al*, 1994; Hidalgo *et al*, 1997; International Germ Cell Consensus Classification, 1997; Ganjoo *et al*, 2000). High-dose chemotherapy with autologous peripheral blood stem cell transplantation (HD-CT) is used as a therapeutic option with acceptable toxicity in patients with relapsed GCT, and has also been investigated as first-line therapy in patients fulfilling ‘intermediate’ or ‘poor prognosis’ criteria according to the IGCCCG classification (Motzer *et al*, 1993,^1997^; Bokemeyer *et al*, 1998). A retrospective matched-pair analysis demonstrated that first-line HD-CT is associated with a potential survival benefit of approximately 15% at 2 years in ‘poor prognosis’ patients compared to standard-dose cisplatin-based chemotherapy (Bokemeyer *et al*, 1999). This report summarises our experience with HD-CT in patients with mediastinal nonseminomatous GCT. Those patients were included into a German multicentre trial using HD-VIP regimen as first-line treatment in patients fulfilling either ‘advanced disease’ criteria according to the Indiana classification or IGCCCG ‘poor prognosis’ criteria since its introduction in 1995 (Birch *et al*, 1986; International Germ Cell Consensus Classification, 1997).

## PATIENTS AND METHODS

**Figure 1:**
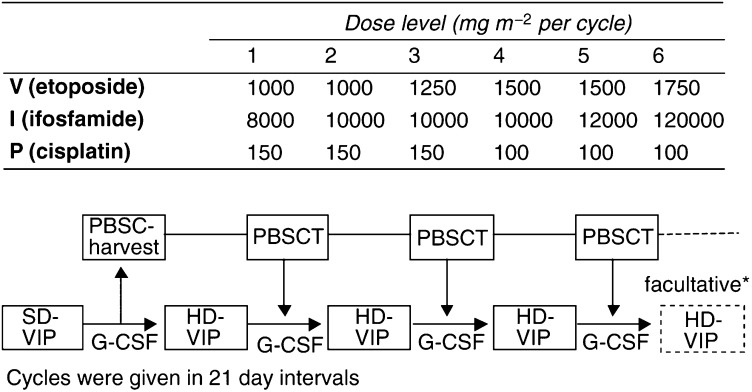
Design of sequential HD-VIP with PBSCT. SD=standard dose; HD=high dose; G-CSF=granulocyte-colony-stimulating factor; PBSC(T)=autologous peripheral blood stem cell (transplantation). ^*^Five patients received a 4th HD-VIP cycle because of declining, but not completely normalised, tumour maker concentrations.

From January 1993 to July 1998, 28 patients with primary mediastinal nonseminomatous GCT were treated with sequential first-line HD-VIP chemotherapy followed by autologous peripheral blood stem cell transplantation within a German prospective, multicentre trial. Eligibility criteria included nonseminomatous histology, any primary site, ‘advanced disease’ according to the Indiana University Criteria (Birch *et al*, 1986) or ‘poor prognosis’ according to the *IGCCCG* (International Germ Cell Consensus Classification, 1997), adequate kidney function (creatinine clearance >60 ml min^−1^) and liver function (bilirubin <1.5-fold upper normal limit, liver enzymes <three-fold upper normal limit), no prior chemotherapy as well as informed consent. The local ethical committee had approved the study. Treatment consisted of one cycle of standard-dose VIP-chemotherapy (cisplatin 20 mg m^−2^, etoposide 75 mg m^−2^, ifosfamide 1200 mg m^−2^, daily for 5 days) plus granulocyte-colony stimulating factor (G-CSF) 5 *μ*g kg^−1^ starting at day 7 followed by peripheral blood stem cell collection and subsequently three sequential cycles of HD-VIP chemotherapy. Another HD-VIP cycle was allowed in case of declining but not completely normalised tumour marker concentrations. The dosages of the high-dose cycles were escalated over six different patient cohorts. No intraindividual dose escalation was performed. Data on the treatment protocol have been reported elsewhere (Bokemeyer *et al*, 1998). The dosages applied ranged from 200 to 350 mg m^−2^ for etoposide, 1.6–2.4 gm^−2^ ifosfamide and cisplatin 20 (levels 4–6) to 30 mg m^−2^ (levels 1–3) daily for five consecutive days every 3 week. All patients received autologous peripheral blood stem cell transplantation (at least 1 × 10^6^ CD 34+ cells kg^−1^ body weight) at day 7 after every 5-day HD-VIP application as well as 5 *μ*g kg^−1^ body weight G-CSF (filgrastim) support subcutaneously starting 24 h after completion of chemotherapy (for details see Figure 1

). All patients have been analysed on an intention-to-treat principle. An extragonadal mediastinal GCT by definition required the absence of testicular abnormalities on physical examination and ultrasonography. In case of testicular abnormalities, biopsy was performed to exclude invasive testicular germ cell cancer. Testicular intraepithelial neoplasia or a scar found at biopsy was not a cause for exclusion from study. Resection of residual tumour masses after chemotherapy was planned, if technically feasible, for all patients with marker negative partial remission (PR) or in patients with still decreasing but not completely normalised markers and partial radiological responses. Resected specimens were categorised according to the following criteria: necrosis referred to findings of necrotic debris only in the resected specimen. Differentiated teratoma referred to the finding of mature teratoma in the absence of either malignant transformation or GCT such as embryonal carcinoma, yolk sac carcinoma, choriocarcinoma or seminoma. Any of the latter histological findings were considered as vital tumour as well as teratoma with malignant transformation. In case of a complete resection of all residual masses, patients were categorised depending on the histological results as no evidence of disease (NED) (either vital tumour, differentiated teratoma or necrosis). However, in four patients who had attained serologic complete remission (CR) but had persistent minor radiographic abnormalities, individual investigators have chosen to observe such patients without surgery because of nonresectability. Those patients were formally coded as PR in the follow-up status if their residual abnormalities remained stable or decreased on imaging studies over a 1-year period.

A CR was defined as the complete disappearance of all clinical, radiological and biochemical evidence of disease after chemotherapy. Marker negative partial remission (PRm-) was assumed in patients with any decrease in the sum of the products of diameters of measurable lesions and normalisation of tumour markers. Progressive disease (PD) was defined as either residual lesions increasing in size or occurrence of new lesions and/or elevation of tumour markers at repeated controls.

### Statistical analysis

Statistical analysis was performed using SPSS (SPSS for Windows 10.0. software SPPS Inc., Chicago, IL, USA). For all living patients, the status as of January 2002 was obtained. The overall survival (OS) calculation used death due to any reason as the end point. Various patients' characteristics such as, categorial variables, extent of disease at diagnosis, evidence of bone, lung, liver, brain, lymph node involvement, age grouping or tumour marker grouping (according to the IGCCCG classification) were investigated as potential prognostic factors by univariate analysis. For the survival time, the proportion of survivors was estimated by the Kaplan–Meier method (Kaplan and Meier, 1958), and the log-rank test was used for comparison. For ordered categorial variables, the log-rank test for trends was used. All factors with a *P*-value <0.05 were considered as significant. All reported *P*-values were two-sided.

The survival data of patients with high-dose chemotherapy have been compared with the cohort of patients receiving cisplatin-based conventional dose chemotherapy available from the *International Extragonadal Germ Cell Tumour Study Group*, which comprised individual treatment data on 635 patients with extragonadal GCT from 11 European and US cancer centres (Bokemeyer *et al*, 2002; Hartmann *et al*, 2002). A small proportion of patients with conventional dose chemotherapy received double-dose cisplatin, which had no impact on efficacy in a randomised, prospective intergroup study (Nichols *et al*, 1991).

## RESULTS

### Patients' characteristics

Twenty-eight patients with primary mediastinal nonseminomatous GCT were treated within the HD-VIP protocol from 1993 to 1998. The median age was 32 years (range, 20–50). A detailed list of the patients' characteristics is given in Table 1

**Table 1:** Characteristics of 28 patients with nonseminomatous mediastinal GCT

**Characteristics**	No. of pts	**%**
Median age – years (range)	32 (20–50)	–
Histology		
Nonseminomatous GCT^a^	28^b^	100
Teratocarcinoma	4	14
Endodermal sinus tumour	8	29
Choriocarcinoma	8	29
Embryonal Cell	1	4
Serologic diagnosis only^c^	1	4
other GCT histology	6	21
Elevated serum tumour markers		
*α*-fetoprotein	17	61
Median (range) in ng ml^−1^	912 (1 to 31.114)	
Human chorionic gonadotropin	10	36
Median (range) in mIU ml^−1^	34 (0.9 to 300.000)	
Lactate dehydrogenase	12	43
Median (range) in IU l^−1^	360 (113 to 2.682)	
Extent of disease		
Localised mediastinal mass	10	32
Additional lung/pleural involvement	7	25
Visceral metastases	11	39
Sites of metastases		
Bone	5	18
Lung	17	61
Liver	7	25
CNS	3	11
LN	3	11
Other	5	18
Previous chemotherapy	–	–

a GCT=germ cell tumour;

b A total of 12 of 28 patients have been included in Bokemeyer *et al* (1998);

c Patient had a mediastinal mass without histologic diagnosis of the tumour and elevation of tumour marker concentrations *β*-HCG and/or AFP indicating a mediastinal nonseminomatous GCT.

. Due to the presence of the primary mediastinal GCT, all patients were classified as ‘poor prognosis’ testicular germ cell cancer according to the IGCCCG classification. Even without considering the primary tumour site, 18 patients (64%) fulfilled ‘poor prognosis’ criteria either because of the additional presence of visceral metastases or ‘poor marker’ status. Serum concentrations of the tumour markers *α*-fetoprotein, *β*-human chorionic gonadotropin and lactate dehydrogenase were elevated in 61, 36 and 43% of the 28 patients, respectively. Ten patients had localized disease confined to the mediastinum (36%), seven (25%) had additional thoracic involvement and 11 patients (39%) had evidence of visceral disease.

### Treatment side effects

A total of 89 high-dose cycles with a median number of 3 (range, 3–4) per patient were given to the 28 patients with mediastinal primary tumours resulting in a median duration of WHO grade IV granulo- and thrombocytopenia of 6 and 4 days. Median time to haematologic recovery from the start of treatment was at days 15 and 16, respectively. WHO grade III/IV mucositis/enteritis occurred in nine (32%) patients. Nine (32%) patients developed grade III/IV fever/infection. No toxic death occurred. Chemotherapy-related neurotoxicity grade I/II was observed in five (18%) patients.

### Response to treatment

Median follow-up duration was 43 months (range, 7–113) for all patients and 52 months (range, 22–113) for surviving patients. Follow-up data and response evaluation are summarised in Table 2

**Table 2:** Response to treatment and current follow-up status

	**No. of pts**	%
Response to treatment^a^		
CR/NED_necrosis_	11 (4^b^/7)	39 (14/25)
NED_vital tumour_	7^c^	25
NED_teratoma_	1	4
PRm−	4	14
PD	5	18
Follow-up status		
NED	17^d^	61
Continuously NED	16	57
PR (>1 year)	1	4
DoD	8	29
Dead due to haematologic disorder	2^e^	7
Estimated OS	%	CI95%
At 2 years	68	51–85
At 5 years	64	46–82
Estimated PFS		
At 2 years	64	47–82
At 5 years	56	37–75

a Evaluation of response based on CT scans and determination of serum tumour marker concentrations.

b Including one patient with consolidation CNS radiation for CNS metastases.

c One patient with non-germ cell tumour elements.

d One patient after resection of mature teratoma at relapse.

e One patient with simultaneous relapse of GCT (after achievement of PRm−) and development of haematologic disorder (myelodysplasia with abnormal megakaryocyctes).

CR=complete remission after chemotherapy alone; NED_vital tumour/teratoma/necrosis_=no evidence of disease after complete resection of vital tumour or differentiated teratoma; PR=partial remission; PD=Progressive disease; DoD=dead of disease; CI=confidence interval; OS=overall survival; PFS=progression-free survival; pts=patients.

. Nineteen of 28 patients (68%) obtained a disease-free status after treatment. Of the 19 patients with no evidence of disease after treatment, 16 are continuously in CR and in addition, one of four patients with PRm- after HD-VIP is currently alive. Two patients have relapsed from CR with recurrent GCT (*n*=1) and teratoma (*n*=1). Relapses occurred after 32 and 43 months after treatment. The patient who had developed teratoma remained alive and disease free 37 months after mediastinal resection and atypical right upper lobectomy. Three of four patients relapsed after achievement of a marker normalised PR at 2, 5 and 10 months after completion of chemotherapy. Two patients developed haematologic disorders, one an acute myelogenous leukaemia and the other a myelodysplasia with abnormal megakaryocytes, 12 and 18 months after initial therapy. Both died shortly after the occurrence of the haematologic disorder. Patients, who did not attain a CR to HD-VIP, received different second-line chemotherapy regimens including VeIP (Loehrer *et al*, 1998) Loehrer et al, 2001 not cited in ref. List, TIP (Rick *et al*, 2001), continuation of standard VIP and oral etoposide; however, none of the patients responded to salvage treatment. Neither the median progression-free survival (PFS) nor the median OS has been reached. The estimated 2- and 5-year PFS rates are 64% (95 % CI, 47–82%) and 56% (95% CI, 37–75%), and the estimated OS rates are 68% (95% CI, 51–85%) at 2 years and 64% (95% CI, 46–82%) at 5 years.

### Univariate analysis on prognostic factors for survival

Results are summarised in Table 3

**Table 3:** Univariate analysis of patients' and treatment characteristics for their influence on OS in nonseminomatous mediastinal GCT

**Characteristics**	**No. of pts (%)**	**Calculated 5-year OS rate (%)**	***P***	**Calculated 5-year PFS rate (%)**	***P***
*Extent of disease*					
Mediastinum only	10 (32)	90		90	
Lung/pleural	7 (25)	57		29	
Visceral disease	11 (36)	45	0.08	45	0.02
*Bone metastases*					
No	23 (82)	64		59	
Yes	5 (18)	60	0.87	60	0.93
*Lung metastases*					
No	11 (39)	89		89	
Yes	17 (61)	53	0.23	41	0.03
*CNS metastases*					
No	25 (89)	58		54	
Yes	3 (11)	100	0.22	100	0.18
*Liver metastases*					
No	21 (75)	80		75	
Yes	7 (25)	14	<0.01	14	<0.01
*Lymph nodes*^1^					
No	25 (89)	66		63	
Yes	3 (11)	33	0.12	33	0.38
Age					
20–25	6 (21)	0		0	
26–30	7 (25)	86		86	
31–35	7 (25)	57		43	
36–40	5 (18)	80		80	
>40	3 (11)	100	0.02	67	0.27
Tumour marker status^2^					
AFP					
Good/intermediate	18 (64)	67		56	
Poor	7 (25)	57	0.65	57	0.87
*β*-HCG					
Good/intermediate	20 (71)	65		60	
Poor	5 (18)	60	0.86	40	0.37
LDH					
Good/intermediate	24 (86)	67		58	
Poor	1 (4)	0	0.14	0	0.29

1 Additional regional lymph node involvement outside of the midline.

2 According to International germ cell consensus classification, 1997.

*β*-HCG=beta human chorionic gonadotropin; LDH=lactate dehydrogenase; LN=lymph nodes; OS=overall survival; PFS=progression-free survival; pts=patients.

. A significant inferior PFS was found for patients who had a disease extending beyond the mediastinum with evidence of liver or pulmonary metastases. Disease confined to the mediastinum indicated a superior outcome. The presence of liver metastases was identified to worsen OS. Elevation of tumour marker concentrations and categorisation into ‘good/intermediate’– *vs* ‘poor’ – marker according to the IGCCCG did not influence either PFS or OS.

### Comparison to standard-dose cisplatin-containing treatment programmes

Two hundred fifty three patients with mediastinal GCT, who had received cisplatin-based conventional chemotherapy between 1979 and 1996, were available from the database of the *International Extragonadal Germ Cell Tumour Study Group* comprising 635 patients with extragonadal GCT from 11 cancer centres in the US and Europe (Bokemeyer *et al*, 2002; Hartmann *et al*, 2002). The results of patients with nonseminomatous mediastinal GCT undergoing standard-dose regimen were compared with our experience in patients with dose-intensive chemotherapy according to the HD-VIP protocol (Table 4

**Table 4:** Comparison of trials in patients with primary mediastinal nonseminomatous GST including conventional cisplatin-based regimen and high-dose chemotherapy

	**Conventional dose**^a^		**HD-VIP**	
Variable	**No. of pts**	**%**	**No. of pts**	**%**
Patients	253		28	
CR/PRm- (assessable)	157 (244)	45	17 (28)	61
Status				
NED	114	45	17	61
AwD	11	4	1	4
Dead	121	48	10	36
Lost to follow-up	9	4	–	–
Median 5-year PFS rate (%)	42		56	
Median 5-year OS rate (%)	46		64	
Median PFS (months)	15		Not reached	
Median OS (months)	40		Not reached	

HD=high dose; CR=complete remission; PRm-=marker normalized partial remission; NED=no evidence of disease; AwD=alive with disease; PFS=progression-free survival; OS=overall survival; pts=patients.

a Data about patients receiving standard cisplatin chemotherapy are derived from the database of the International Extragonadal Germ Cell Tumour Study Group (in part described in Bokemeyer *et al*, 2002, Hartmann *et al*, 2002).

, Figures 2A,B

**Figure 2:**
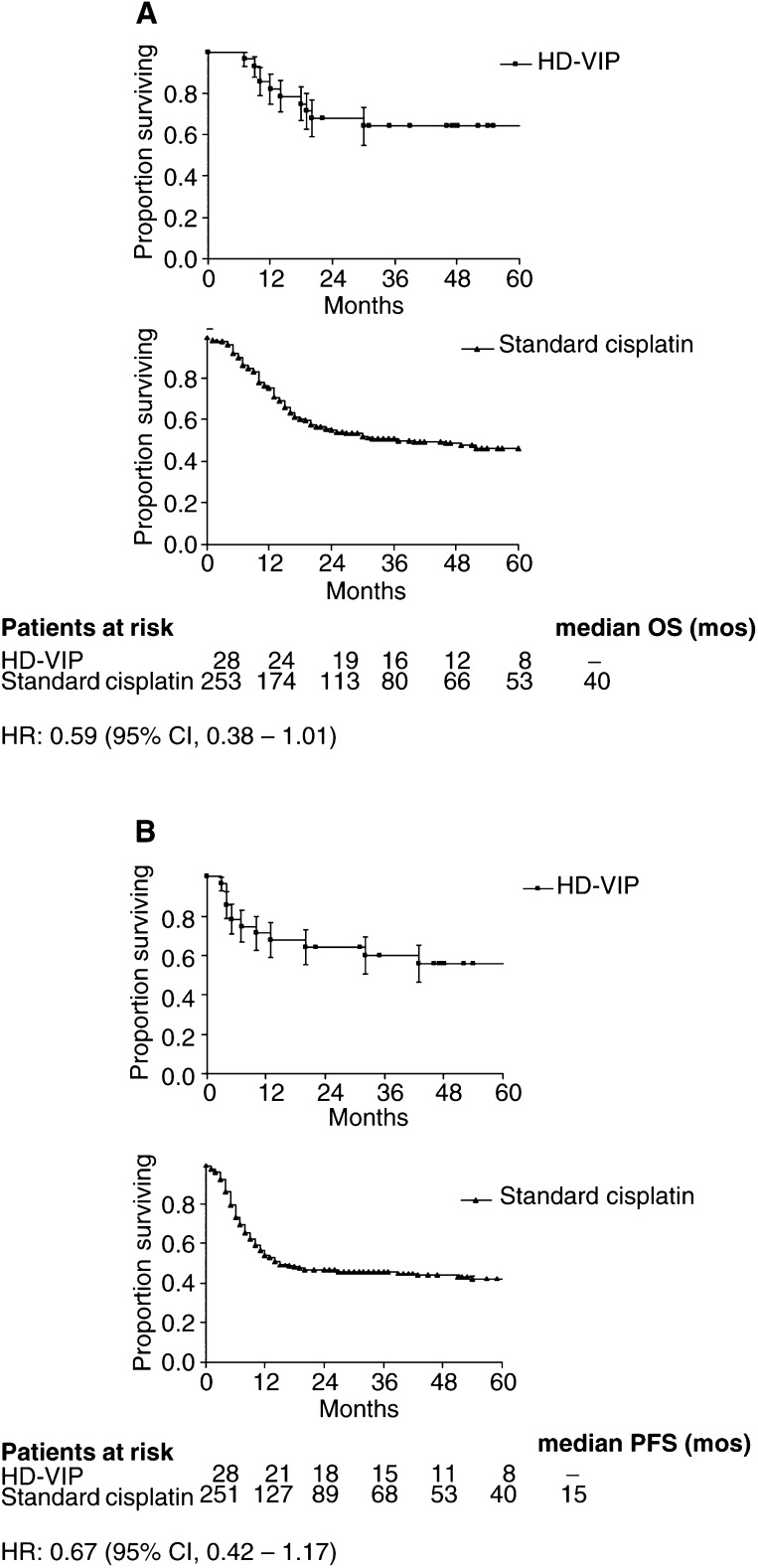
Overall survival (**A**) and PFS (**B**) for patients with primary mediastinal nonseminoma treated on programmes that included either high-dose chemotherapy (HD-VIP) or conventional dose cisplatin-based combination chemotherapy (date about patients receiving standard cisplatin chemotherapy are derived from the database of the International Extragonadal Germ Cell Tumour Study Group (in part described in Bokemeyer *et al*, 2002; Hartmann *et al*, 2002). mos= months

). There was a higher 5-year OS rate with dose-intensive therapy compared to standard-dose cisplatin protocols of 64% *vs* 46% as well as 57% *vs* 42% for PFS rates at 5 years. The corresponding hazard ratios for PFS and OS in patients receiving HD-VIP compared to standard cisplatin chemotherapy were 0.67 (95% CI, 0.42 – 1.17) and 0.59 (95% CI, 0.38 – 1.01), respectively.

## DISCUSSION

Although the principles of management of patients with mediastinal nonseminomatous GCT parallel those of metastatic nonseminomatous testicular GCT, patients with mediastinal primaries clearly have a worse prognosis compared to patients with gonadal GCT. Accordingly, the presence of a mediastinal primary tumour in patients with nonseminomatous GCT was identified as a major adverse prognostic factor by the IGCCCG. Such patients appear to represent a clinically and biologically distinct disease entity (Nichols *et al*, 1985,^1987^,1990a; Hartmann *et al*, 2000b,2001a,2001b), associated with lower complete response rates to chemotherapy, high rates of relapse and more frequent failures to salvage chemotherapy (Saxman *et al*, 1994, Loehrer *et al*, 1998; Hartmann *et al*, 2001a). Historically, the incorporation of cisplatin into chemotherapy regimens has yielded survival rates of approximately 30–40% in retrospective analyses (Garnick *et al*, 1983; Logothetis *et al*, 1985; Delgado *et al*, 1993; Dulmet *et al*, 1993; Toner *et al*, 1991; International germ cell consensus classification, 1997). Two prospective series based on 31 and 41 patients reported a long-term survival rate of >50% following conventional cisplatin-based chemotherapy plus secondary surgery (Nichols *et al*, 1990b, Bukowski *et al*, 1993). The database of the *International Extragonadal Germ Cell Tumour Study Group* contains 287 patients with primary mediastinal tumour location and nonseminomatous histology from 11 Cancer Centres (Bokemeyer *et al*, 2002; Hartmann *et al*, 2002). Of these patients, 253 have been treated with conventional cisplatin-based chemotherapy. The 2- and 5-year OS rates of those patients were 55 and 46%, and 47 and 42% for PFS.

The analysis of the *International Extragonadal Germ Cell Tumour Study Group* demonstrated that patients with primary mediastinal GCT have a heterogeneous prognosis depending on patients' characteristics (Hartmann *et al*, 2002). Patients having localised mediastinal disease without elevation of HCG at initial diagnosis and who were of young age, revealed a favourable survival (OS 83% at 2 years) compared to patients presenting with adverse prognostic variables such as presence of visceral metastases or of lung metastases (OS 34 or 42% at 2 years). One conclusion from this investigation is that patients with localised disease confined to the mediastinum have an acceptable prognosis when treated by a multidisciplinary approach with aggressive surgery of the residual mediastinal tumour mass.

However, more than half of the patients with mediastinal nonseminomatous GCT failed to achieve a durable response to conventional cisplatin-based chemotherapy. The success of carboplatin/etoposide- (CE) or carboplatin/etoposide/cyclophosphamide- (CEC) containing high-dose chemotherapy with autologous peripheral blood stem cell transplantation (HD-CT) in the treatment of patients with relapsed disease led to its investigation as initial first-line treatment in patients fulfilling ‘poor prognosis’ criteria. With this approach as initial treatment, the time to blood count recovery is shorter, toxicity is reduced, and further dose intensification is feasible compared with its use in heavily pretreated patients (Motzer *et al*, 1996). Two consecutive trials conducted by investigators at the MSKCC suggested a therapeutic benefit for HD-CT when used as first-line treatment for ‘poor prognosis’ patients (Motzer *et al*, 1993,^1997^). The long-term survival rates for all ‘poor prognosis’ patients have been 54 and 67% after HD-CT with the CE- or CEC-regimen. These trials included single patients with primary mediastinal nonseminomatous GCT; however, no detailed data on response and outcome for this subgroup of patients due to the small numbers were reported.

The use of initial dose intensification in patients with nonseminomatous mediastinal GCT appears reasonable because the results with salvage treatment, even including HD-CT, have been rather disappointing. It appears that more than 90% of patients with relapsed mediastinal nonseminoma fail to obtain a durable, complete remission in the salvage setting (Josefsen *et al*, 1993; Saxman *et al*, 1994; Loehrer *et al*, 1998; Hartmann *et al*, 2001a).

The current series on the use of intensive first-line HD-VIP for patients with primary mediastinal nonseminoma indicates the feasibility of this approach, and a 15–20% absolute survival improvement for these patients receiving sequential HD-VIP as initial treatment compared to the use of conventional induction chemotherapy might be achievable. Calculation of the hazard ratios portended a 33% and 41%-risk reduction in recurrence and death probability for patients treated with HD-VIP compared to standard cisplatin chemotherapy. However, this is based on a small sample size in the high-dose chemotherapy group. The comparison of high-dose and standard-dose chemotherapy was not performed as a prospective randomised trial, and therefore patient selection and stage migration might be an issue since both groups of patients had been treated in a different time period.

Prognostic factors for patients receiving HD-VIP in the univariate analysis have been similar to those in the *International Extragonadal Germ Cell Tumour Study Group* database – localised mediastinal disease *vs* visceral disease, for example, liver, bone, CNS metastases and lung/pleural involvement. The OS rates at 2 years achieved in this investigation compared to the expected rates according to the prognostic index of the *International Extragonadal Germ Cell Tumour Study Group* for mediastinal nonseminomas have been 100 *vs* 65% for patients with localised disease, 57 *vs* 42% for disease confined to the thorax (patients with lung or pleural metastases) and 45 *vs* 34% for patients with presence of visceral metastases (Hartmann *et al*, 2002), suggesting that the results observed in this investigation of dose-intensified HD-VIP chemotherapy might not be achieved due to a selection of patients possessing more favourable prognostic characteristics. The only way to truly ascertain the difference between standard- and high-dose chemotherapy is to perform a randomised trial. An ongoing phase III trial has been initiated in the US to define the role of HD-CT including high-dose carboplatin instead of cisplatin in ‘intermediate’ and ‘poor prognosis’ patients. This trial also includes patients with primary mediastinal nonseminoma. However, because of the rarity of mediastinal location within the group of poor prognosis patients, this trial will only include approximately 12–18 patients with primary mediastinal GCT in each arm that will not allow a sufficient subgroup analysis.

Overall, the survival rates in patients with mediastinal GCT have been improved over the years – particularly due to aggressive postchemotherapy surgery of residual mediastinal masses – now approaching the rate which is achieved in patients with ‘poor prognosis’ metastatic disease according to the IGCCCG classification (International Germ Cell Consensus Classification, 1997). More than half of the patients in the current series have undergone a multidisciplinary treatment approach including aggressive postchemotherapy surgery and the use of consolidation radiation of the brain in one patient. As in patients with metastatic gonadal GCT, radical surgical resection of residual masses after first-line chemotherapy is indicated, whenever technically possible either as a one-stage or as a sequential procedure (Kay *et al*, 1987; Wright *et al*, 1990; Nichols *et al*, 1990b; Hartmann *et al*, 1997; Ganjoo *et al*, 2000; Vuky *et al*, 2001). In this series, all 10 patients with disease confined to the mediastinum regardless of other prognostic factors that are relevant for metastastic gonadal GCT, for example, tumour marker status, are disease-free. One of these patients died due to a haematologic disorder without evidence of GCT relapse.

As described before, there is approximately a 6% risk to develop a haematologic disorder in patients with primary mediastinal nonseminoma, which represents a biological phenomenon not related to chemotherapy (Nichols *et al*, 1985,1990a; Hartmann *et al*, 2000b). The haematologic malignancies have a very aggressive clinical course with patients either dying before treatment, not responding to antileukaemic therapy, or achieving remissions of very short duration. In the current investigation, two patients died due to haematologic disorders 12 and 18 months after completion of GCT treatment.

Investigational approaches in progress explore whether the outcome of patients with mediastinal primary GCT can be further improved with the use of first-line sequential HD-CT or with the addition of further active drugs to the HD-VIP regimen (Hartmann *et al*, 2001c). In order to improve the prognosis of patients with mediastial GCT, it appears mandatory to include these patients into controlled clinical trials at experienced centres.
